# Molecular Epidemiological Characteristics and Genetic Evolution of Bluetongue Virus in Yunnan Province, China

**DOI:** 10.3390/microorganisms14081697

**Published:** 2026-08-02

**Authors:** Zhenxing Yang, Yuwen He, Yongkang Li, Jinxin Meng, Nan Li, Susheng Li, Haisheng Miao, Jianling Song

**Affiliations:** 1 Yunnan Tropical and Subtropical Animal Viral Disease Laboratory, Yunnan Animal Science and Veterinary Institute, Kunming 650224, China; s300yn@163.com (Z.Y.); heyuwen1117@163.com (Y.H.); mengjinxin1989@163.com (J.M.); linan691@126.com (N.L.); sushenglee@163.com (S.L.); 2 College of Veterinary Medicine, Yunnan Agricultural University, Kunming 650201, China; 13238694970@163.com

**Keywords:** bluetongue virus, Culicoides midges, molecular epidemiology, genetic evolution

## Abstract

Bluetongue is an insect-borne disease of ruminants caused by the Bluetongue virus (BTV). It is primarily transmitted through the bites of Culicoides midges and is widely distributed in tropical, subtropical, and temperate regions around the world. Due to its favorable geographical location and abundant population of BTV vector midges, Yunnan Province, China, has become a key region for research on BTV diversity. However, there are gaps in research on the genetic evolution of BTV in this region. Between January 2024 and December 2025, Culicoides midges were collected from three border counties in Yunnan Province. Researchers isolated BTV strains and sequenced the full-length segment 2 (Seg-2) gene. Subsequently, phylogenetic analysis, Bayesian evolutionary rate analysis, population dynamics analysis, and phylogeographic analysis were performed utilizing Seg-2 sequence data sourced from the NCBI database. Four BTV serotypes (BTV-1, BTV-4, BTV-5, and BTV-16) were identified. The estimated average evolutionary rate varied from 2.88 × 10^−4^ to 4.26 × 10^−3^ substitutions per site per year, with ancestral origins dating back to the period between 1872 and 1988. The effective population size displayed significant fluctuations over time. Phylogeographic reconstructions indicated that the pathogen entered Yunnan through various transcontinental transmission pathways, originating from Europe, South Asia, Oceania, and East Asia, with evidence of reciprocal transmission between Africa, Europe, and West Asia. The BTV serotypes circulating in Yunnan Province are highly diverse, and the population dynamics of different serotypes exhibit significant heterogeneity. Yunnan is simultaneously exposed to BTV introductions from multiple directions, including Europe, South Asia, Oceania, and East Asia.

## 1. Introduction

Bluetongue (BT) is an infectious disease affecting domestic and wild ruminants. It is caused by the bluetongue virus (BTV), which belongs to the genus Orbivirus within the family Reoviridae [1,2,3]. The virus is primarily transmitted through the bites of Culicoides midges in the family Ceratopognidae, which feed on susceptible hosts [4,5]. Additional transmission routes include sexual transmission via semen, contact transmission, and oral transmission [6,7]. BT outbreaks exhibit a distinct seasonal pattern, typically occurring in late summer and fall [8]. These outbreaks are observed across tropical, subtropical, and temperate regions. The World Organisation for Animal Health (WOAH) classifies BTV as a multispecies disease [9]. Outbreaks of disease due to pathogenic BTV strains can result in significant economic losses.

BTV is a non-enveloped virus characterized by a 10-segment double-stranded RNA (dsRNA) genome, which is enclosed within a three-layered icosahedral capsid [10,11]. The genome segments vary in size from 3954 to 822 bp and are organized in descending order of molecular weight, from Seg-1 to Seg-10 [12]. The BTV genome encodes seven structural proteins (VP1–VP7) and four non-structural proteins (NS1–NS4) [11,13,14]. Variations in the sequence of genome segment 2 (Seg-2) and its corresponding protein VP2 have led to the identification of 27 BTV serotypes [15,16,17].

BTV was initially identified in South Africa in 1876, coinciding with the introduction of domesticated sheep from Europe to the region [18]. As global vector distribution expanded and animal movement increased through trade, the disease has emerged in various regions, including Asia, Europe, Australia, and North America, thus being recognized as a transcontinental disease. In China, BTV was first detected in 1979 in Shizong County, Yunnan Province [19]. Since that time, 16 serotypes (BTV 1, 2, 3, 4, 5, 7, 9, 11, 12, 14, 15, 16, 21, 23, 24, and 29) have been identified [20,21].

Most previous studies on BTV in Yunnan Province have focused primarily on serotype identification, resulting in a gap in research on the reconstruction of BTV’s evolutionary history. This study involved the isolation of BTV and sequencing of the Seg2 gene from Culicoides midges captured in Yunnan Province from January 2024 to December 2025. Additionally, a system dynamics analysis was conducted using all publicly available seg2 gene sequences worldwide to infer the geographic origin of BTV in Yunnan Province and its intercontinental transmission routes. The findings will help elucidate the transmission characteristics of BTV in this region, provide a basis for local BTV molecular surveillance efforts, and enhance their effectiveness, ultimately enabling effective control of outbreaks caused by emerging and endemic strains.

## 2. Materials and Methods

### 2.1. Collection and Sorting of Culicoides Midges

Between January 2024 and December 2025, Culicoides midges were collected from cattle shelters in the suburbs of Shuangjiang (99°48′28″ E, 23°30′14″ N), Jinghong (100°51′45″ E, 22°5′20″ N), and Shizong (104°17′28″ E, 24°36′33″ N) Counties, Yunnan Province, using light traps (Wuhan Jixing Environmental Technology Co., Ltd., Wuhan, China) operated from 5 p.m. to 7 a.m. These locations are situated near the borders with Myanmar, Laos, and Vietnam, respectively. Groups of one hundred Culicoides midges of the same species, collected from a single location, were pooled in a cryopreservation tube, stored in liquid nitrogen, and sent to the laboratory for subsequent analysis.

### 2.2. Viral Genome Extraction and BTV Detection

Homogenized groups of 100 Culicoides midges in 1 mL of ice-cold Modified Eagle’s Medium (MEM) supplemented with 100 U/mL penicillin and 100 µg/mL streptomycin using a tissue grinder. Extracted viral genome from a 50 μL volume of the homogenized solution with the MagMAX™ M-96 Viral RNA Isolation kit and the MagMAX™ Express-96 machine (Ambion^®^, Thermo Fisher Scientific, Waltham, MA, USA). The extracted RNA was denatured at 95 °C, rapidly cooled on ice, and subjected to RT-qPCR with BTV-specific primers (Forward: 5′-TGGAYAAAGCGATGTCAAA-3′, Reverse: 5′-ACATCATCACGAAACGCTTC-3′) and probes (5′-FAM-ARGCTGCATTCGCATCGTACGC-BHQ1-3′) [22]. Mixed 2 μL nucleic acid sample with 18 μL of the reaction solution using the One Step PrimeScript RT-qPCR Kit (Takara Biotechnology Co, Ltd., Dalian, China) following the manufacturer’s protocol.

### 2.3. Reverse Transcription, Amplification, Cloning, and Sequencing of Seg-2 cDNA

The viral genome underwent full-length cDNA amplification (FLAC) following a previously outlined protocol [23,24]. Initially, the viral genome was connected to an anchor primer (5′ p-GACCTCTGAGGATTCTAAAC/iSp9/TCCAGTTTAGAATCC-OH 3′) using T4 RNA ligase. Subsequently, reverse transcription was performed utilizing a PrimeScript™ II Reverse Transcriptase Kit (TaKaRa, Dalian, China). The cDNA was then amplified with a primer (5′-GAGGGATCCAGTTTAGAATCCTCAGAGGTC-3′) designed for cloning purposes, employing the high-fidelity PrimeSTAR^®^ GXL DNA Polymerase (TaKaRa). Following amplification, the cDNA amplicons were purified using the DNA Fragment Purification Kit Version 2.0 (TaKaRa) and inserted into the pTOPO/Blunt vector provided with the Zero Background pTOPO/Blunt Cloning Kit^®^ (Aidlab, Beijing, China). The recombinant plasmids were introduced into Escherichia coli DH5α competent cells. Positive clones were distinguished through PCR using M13 universal primers and subsequently sequenced using an automated ABI 3730xl DNA Sequencer (Applied Biosystems, Waltham, MA, USA).

### 2.4. Global Phylogeny of the BTV Seg-2 Gene

This study included four BTV Seg-2 gene sequences from different serotypes: BTV-1, BTV-4, BTV-5, and BTV-16. Additionally, we obtained all available global Seg-2 gene sequences for these four serotypes from the NCBI Virus database. Exclude sequences with missing sampling location or time information. We ultimately obtained 71 BTV-1, 59 BTV-4, 17 BTV-5, and 80 BTV-16 Seg-2 gene sequences. Multiple sequence alignment of BTV was performed using the MAFFT software (v7.310) [25]. These sequence ends were then trimmed using BioEdit software (v7.2.5) [26]. The BTV phylogenetic ML tree was constructed using IQ-TREE (version 3.1.1), and ModelFinder was used to select the optimal alternative model [27]. Robustness was assessed using 1000 ultra-fast bootstraps (UFBoot) [28]. After inferring the GTR+F+R3, TIM+F+R3, and TN+F+R3 substitution models using AIC, AICc and BIC, respectively, the best-fitting substitution model was selected. Phylogenetic trees were visualized using the online tool Chiplot (https://chiplot.online) [29].

### 2.5. Genetic Evolution and Effective Population Analysis of BTV

A Maximum Clade Confidence (MCC) tree was constructed to assess the genetic evolutionary rate and effective population size of BTV. Bayesian skyline analysis was employed to reconstruct the effective population dynamics of BTV strains. Analysis parameters were set using the BEAST program (v1.10.4) [30]. The nucleotide substitution model utilized was the General Time Reversible (GTR) model, incorporating a Gamma distribution and Invariant Sites (I) to account for rate heterogeneity among sites. The molecular clock model chosen was the Uncorrelated Lognormal Relaxed Clock model [31,32,33]. The Bayesian Skygrid served as the prior model for the population history model to simulate changes in the virus’s effective population size. BEAST (v1.10.4) was executed with an MCMC chain length of 300 million, sampling every 30,000 generations to produce XML files, and leveraging the BEAGLE library for computational acceleration [34]. Parameter convergence and effective sample size (ESS) were evaluated using the Tracer software (v1.7.2). ESS values exceeding 200 indicated adequate sampling and statistical convergence for all parameters [35]. The initial 10% of the log files from the two independent runs were discarded as the burn-in period. Visualization of the (MCC) phylogenetic tree was done using FigTree (v1.4.3), while the effective Bayesian skyline was depicted using the R ggtree (v4.5.2) package.

### 2.6. Bayesian Systematic Geographical Transmission Pathways of BTV

We employed the SpreaD3 software (v0.9.7.1) to conduct spatial diffusion reconstruction on the posterior tree samples produced by the BEAST analysis [36]. The analysis utilized the final tree set (.trees files) from the BEAST run and a trait file with the sampling locations of each viral sequence as inputs. In SpreaD3, we configured key parameters, such as adopting the Bayesian Skygrid as the prior model and opting for the Discrete Diffusion Model to replicate the virus’s migration in geographic space. Subsequently, we utilized ArcGIS Pro (v3.0) to map the transmission routes based on the Bayesian factor and the posterior probability’s magnitude.

## 3. Results

### 3.1. Characteristics of the BTV Phylogenetic Tree

**Figure 1:**
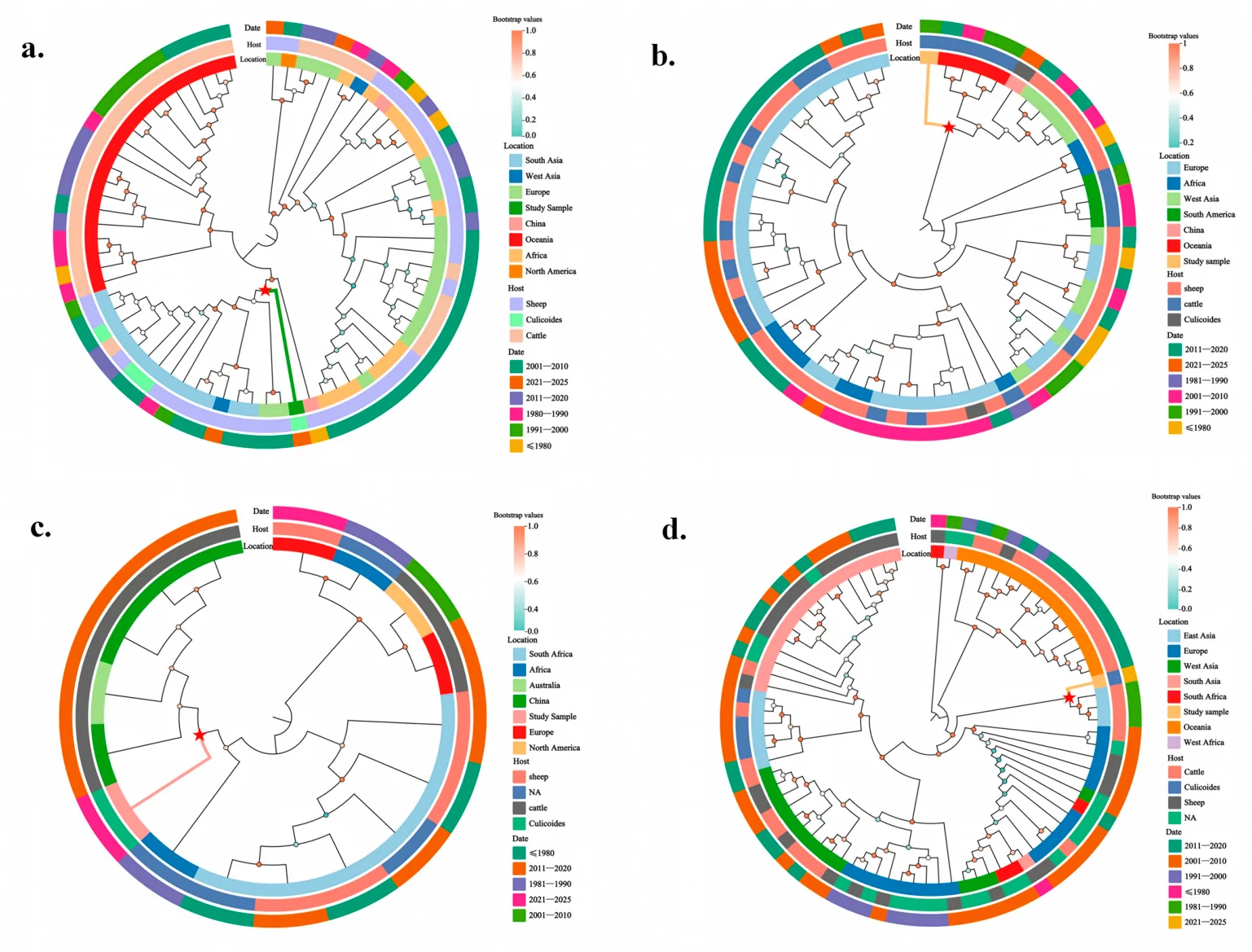
Maximum likelihood phylogenetic tree of the BTV Seg-2 gene in Yunnan Province for 2025. (**a**) BTV-1 ML-Tree. (**b**) BTV-4 ML-Tree. (**c**) BTV-5 ML-Tree. (**d**) BTV-16 ML-Tree. The concentric rings, colored from the innermost to the outermost, represent the sequence’s location, host, and date, respectively. Asterisks of different colors indicate the branch positions of the sample strains.

Between January 2024 and December 2025, Researchers isolated four BTV strains from Culicoides midges collected from cattle herds in the Jinghong, Shizong, and Shuangjiang areas of Yunnan Province. Using the BLAST online tool in the NCBI database, these strains were identified as BTV-1 (Jinghong, 2025), BTV-4 (Shizong, 2024), BTV-5 (Jinghong, 2024), and BTV-16 (Shuangjiang, 2024). Global BTV-1, BTV-4, BTV-5, and BTV-16 strains were simultaneously retrieved; strains lacking sampling time and geographic location information were excluded, and a maximum likelihood (ML) phylogenetic tree of the BTV Seg-2 gene was constructed. Detailed information on the test and reference strains is provided in Supplementary Material Table S1. The BTV-1 sample strain clustered with previously reported strains from Shizong, Yunnan; South Asia (India); West Asia (Israel); and Europe (Spain) (Figure 1a). The BTV-4 sample strain clustered with previously reported strains from Yunnan (1997), Oceania (Australia), and West Asia (Israel) (Figure 1b). The BTV-5 sample strain clustered with previous strains from Yunnan (2012 and 2014), Oceania (Australia), and Africa (South Africa) (Figure 1c). The BTV-16 sample strain clustered with strains from East Asia (Japan) (Figure 1d).

### 3.2. Population Dynamics and Evolutionary History of BTV

**Figure 2:**
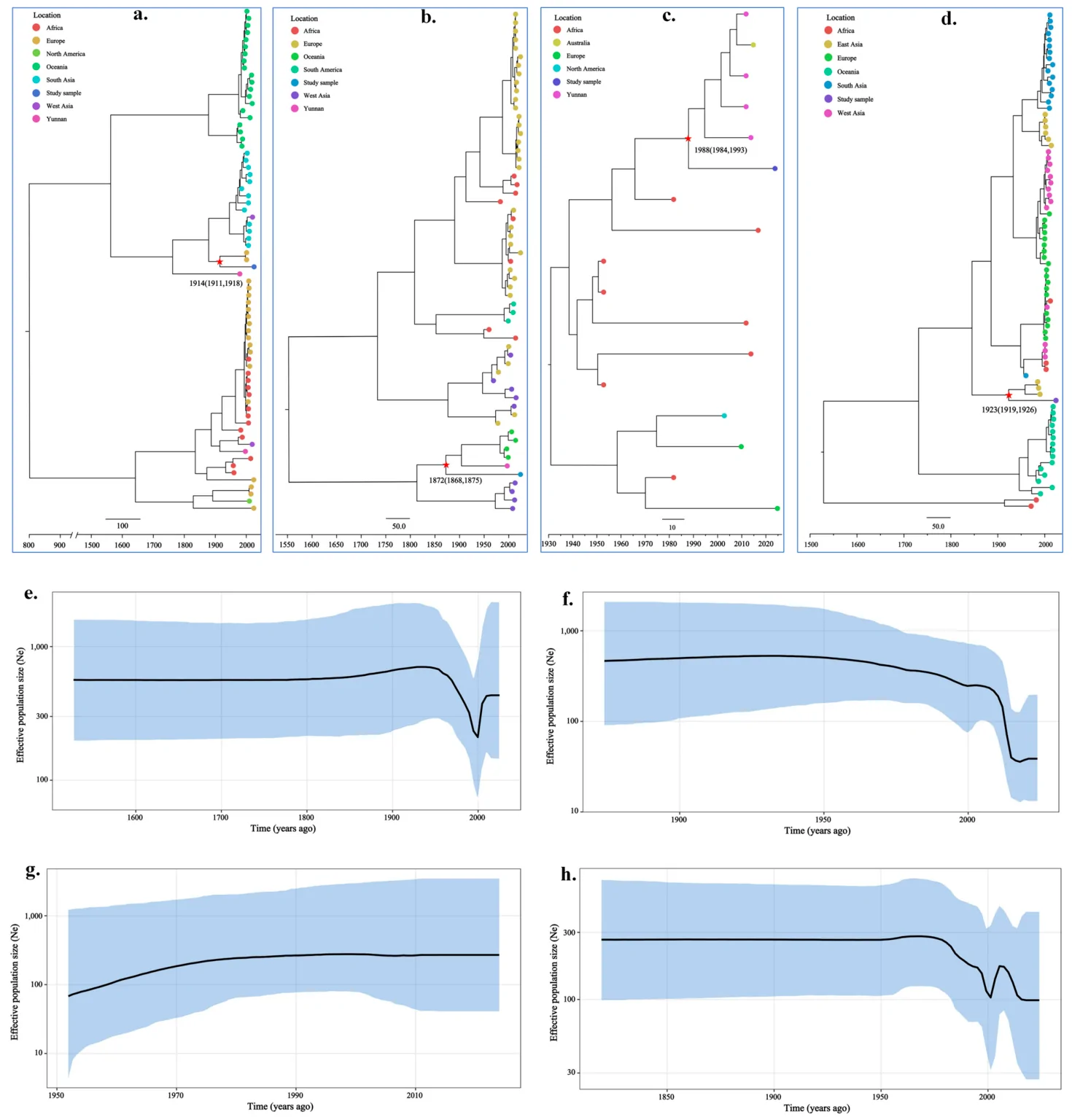
Maximum branch credibility (MCC) trees and Bayesian skyline plots for BTV. (**a**–**d**) MCC trees constructed based on BTV Seg-2 genome sequences. (**a**) BTV-1 MCC tree. (**b**) BTV-4 MCC tree. (**c**) BTV-5 MCC tree. (**d**) BTV-16 MCC tree. Colored dots at the tips of the branches indicate the geographic origin of the samples (see the legend in the figure for the specific color-region correspondence). (**e**–**h**) Bayesian skyline plots showing the change in effective population size (N_e_) over time for BTV-1, BTV-4, BTV-5, and BTV-16, respectively. The solid black line represents the median estimate, and the blue shaded region indicates the 95% highest posterior density (HPD) interval. The *x*-axis represents time (years), and the *y*-axis represents effective population size (N_e_). Asterisks indicate the branch positions of the sample strains.

Maximum-clade-confidence (MCC) trees were constructed separately for BTV-1, BTV-4, BTV-5, and BTV-16. The results showed that the average genetic evolution rate for BTV-1 was 2.88 × 10^−4^ substitutions/site/year (95% HPD: 1.63 × 10^−4^ to 4.25 × 10^−4^ (Figure 2a). The average genetic evolutionary rate for BTV-4 was 4.45 × 10^−4^ substitutions/site/year (95% HPD: 1.44 × 10^−4^ to 8.75 × 10^−4^) (Figure 2b). The average genetic evolution rate of BTV-5 was 4.26 × 10^−3^ substitutions per site per year (95% HPD: 8.13 × 10^−4^ to 8.75 × 10^−3^) (Figure 2c). The average genetic evolutionary rate of BTV-16 was 3.24 × 10^−4^ substitutions/site/year (95% HPD: 1.81 × 10^−4^ to 4.90 × 10^−4^) (Figure 2d). Among them, the ancestral strain of sample BTV-1 originated in 1914 (95% HPD: 1911–1918) (Figure 2a). The ancestral origin of sample strain BTV-4 dates back to 1872 (95% HPD: 1868–1875) (Figure 2b). The ancestral origin of sample strain BTV-5 dates back to 1988 (95% HPD: 1985–1993) (Figure 2c). The ancestral origin of sample strain BTV-16 dates back to 1923 (95% HPD: 1919–1926) (Figure 2d). The effective population size of BTV-1 remained relatively stable until 1953, declined continuously from 1953 to 2000, and then rose steadily after 2000 until reaching a stable state (Figure 2e). The effective population of BTV-4 remained relatively stable until 1960, declined slowly from 1960 to 2010, and then declined sharply after 2010 until reaching a stable state (Figure 2f). The effective population of BTV-5 has remained relatively stable (Figure 2g). The effective population of BTV-16 remained relatively stable until 1980, declined continuously from 1980 to 2000, rose slightly and then sharply from 2000 to 2010, and then declined sharply to a stable state after 2010 (Figure 2h).

### 3.3. Geographic Transmission Pathways of the BTV Bayesian System

**Figure 3:**
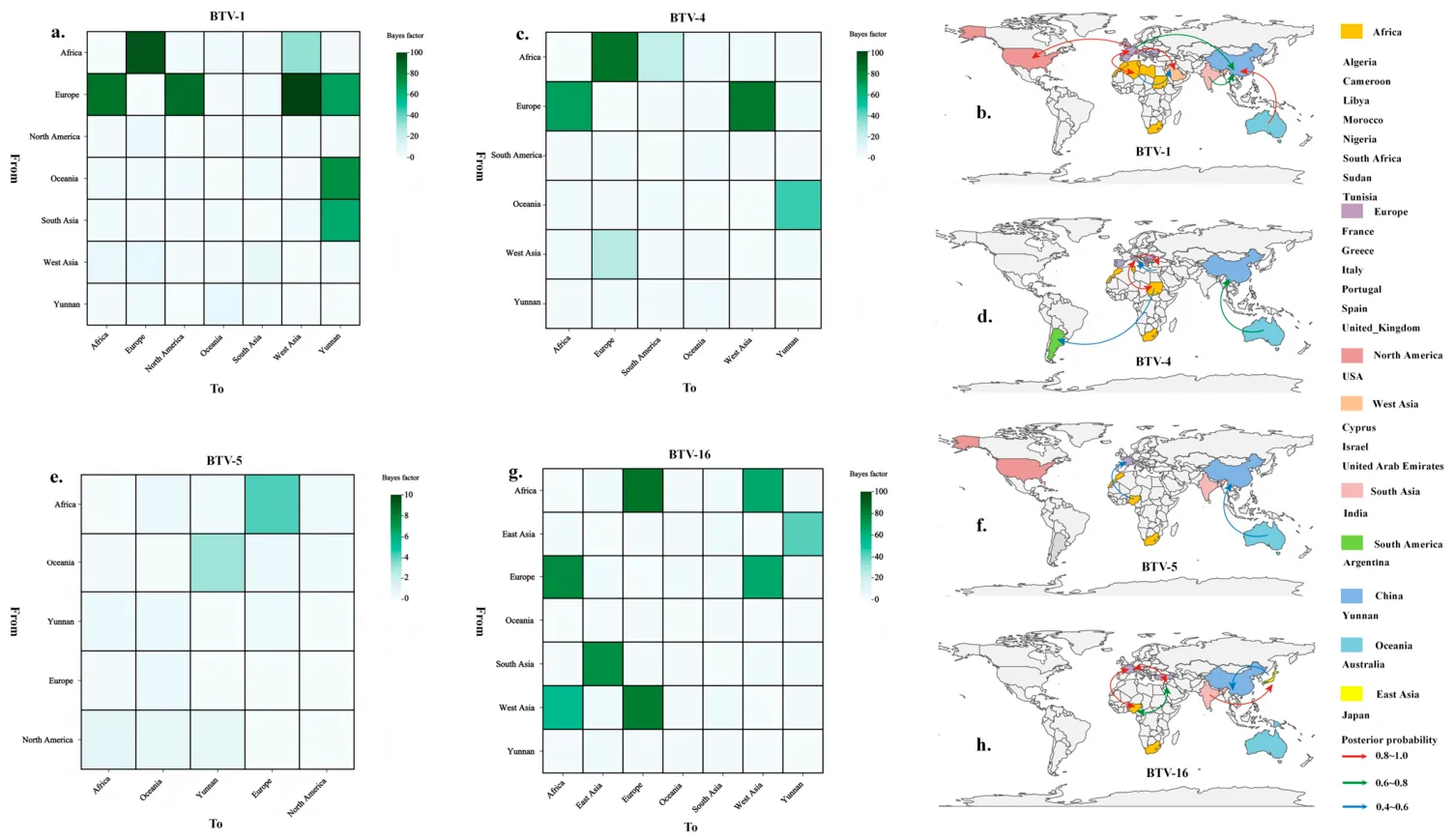
Bayesian phylogeographic analysis inferring global interregional transmission patterns of BTV. (**a**) Heatmap of Bayesian factor (BF) values for BTV-1. (**c**) Heatmap of Bayesian factor (BF) values for BTV-4. (**e**) Heatmap of Bayesian factor (BF) values for BTV-5. (**g**) Heatmap of Bayesian factor (BF) values for BTV-16. Heatmaps of Bayesian factor (BF) values are used to quantify the intensity of viral migration between different locations, with the *x*-axis representing the destination and the *y*-axis representing the origin. (**b**,**d**,**f**,**h**) are spatial projections of the most likely transmission pathways for BTV-1, BTV-4, BTV-5, and BTV-16, respectively. The gradient of arrow colors reflects decreasing posterior probability support (40–100%). The arrows indicate the inferred direction of viral transmission. Rectangles of different colors represent the countries of origin of the gene sequences corresponding to different continents.

To elucidate the global cross-regional transmission patterns of BTV-1, BTV-4, BTV-5, and BTV-16 in this study, SpreaD3 was used to calculate the Bayesian factor values from source to destination to represent transmission intensity (Figure 3a,c,e,g). The transmission pathways for the four subtypes were then visualized in descending order of intensity based on the posterior probability supported by the Bayesian factor (40–100%) (Figure 3b,d,f,h). The transmission pathway of BTV-1 reveals a phenomenon of reciprocal circulation among Africa, Europe, and West Asia (Figure 3b). Concurrently, it was observed that BTV-1 subtypes in Yunnan, China, were primarily introduced from Europe, South Asia (India), and Oceania (Australia) (Figure 3b). The BTV-4 transmission pathways similarly reveal a phenomenon of reciprocal circulation between Europe and West Asia (Figure 3d). BTV-4 in Yunnan, China, is primarily introduced from Oceania (Australia) (Figure 3d). The transmission pathways of BTV-5 indicate that the virus was introduced into Europe from Africa and into Yunnan, China, from Oceania (Australia), although the posterior probability support values are low (40–60%) (Figure 3f). The transmission pathways for BTV-16 similarly reveal a phenomenon of reciprocal circulation between Europe and West Asia (Figure 3h). BTV-16 in Yunnan, China, was primarily introduced from East Asia (Japan), but the posterior probability support was low (40–60%) (Figure 3h).

## 4. Discussion

Bluetongue (BT) is a vector-borne disease of ruminants caused by the bluetongue virus (BTV), primarily transmitted by Culicoides midges. Rising global temperatures have expanded the range and abundance of vectors and their animal reservoirs, further affecting the virus’s development, replication, and survival, thereby posing a potential threat to global livestock production and resulting in significant economic losses.

A study by Carpi et al. on BTV genomic segments indicates that the average substitution rate for the entire BTV genome is approximately 0.5–7.0 × 10^−4^ substitutions per site per year, and that this rate is significantly lower than that of insect-transmitted single-stranded positive-sense RNA viruses [37]. A 30-year study of the evolutionary dynamics of BTV-1 in northern Australia by Boyle et al. [38] similarly reported a rate range of 3.5 × 10^−4^ to 5.3 × 10^−4^ and found that all BTV segments evolved under strong negative selection. The upper limit of the rate estimated in this study (4.26 × 10^−3^) is higher than the estimates from the two aforementioned globally representative studies. This may be because this study analyzed only the single Seg-2 segment, and the time span of sequence sampling was relatively limited. Furthermore, there is significant heterogeneity in the evolutionary rates of different genomic segments of BTV. Carpi et al. [37] clearly pointed out that there are substantial differences in the substitution rates and time to the most recent common ancestor among different segments, and that recombination events have rendered the evolutionary dynamics of each segment highly independent. Alkhamis et al. [31] used a Bayesian system dynamics model to infer that the diversification and spread of BTV worldwide can be traced back more than 1000 years. The significant acceleration in BTV diversification and spread coincides with the rise in intercontinental livestock trade after the 1850s. The time of ancestral origin estimated in this study (beginning in 1872) falls precisely within the time window of accelerated spread via intercontinental livestock trade identified by Alkhamis et al. This temporal alignment provides important insights into the origin of BTV in Yunnan Province, suggesting that it resulted from continuous introductions through channels such as cross-border animal trade during the modern historical period.

This study reconstructed the temporal trends in effective population size for four serotypes using Bayesian skyline plots and found that BTV-16 experienced significant fluctuations in N_e_ from the late 20th century to the early 21st century, whereas BTV-1 and BTV-4 exhibited relatively stable trends. This difference suggests that different serotypes may have distinct epidemiological patterns and vector adaptability within the Yunnan ecosystem. Yunnan’s warm, humid climate supports high Culicoides abundance, and its active cross-border livestock trade facilitates repeated virus introductions—factors that may drive serotype-specific dynamics such as the marked fluctuations observed in BTV-16. It is particularly worth noting that the reference sequences used in this study were derived from strains collected between January 2024 and December 2025, whereas previous epidemiological surveys of BTV in China have shown significant gaps in the data from 2012 to 2014. Yang et al. [39] explicitly reported that China isolated BTV strains of eight serotypes (BTV-1, 2, 3, 4, 9, 12, 15, and 16) from Yunnan and Guangdong provinces between 1996 and 1997, but epidemiological surveys of BTV infection subsequently experienced a prolonged hiatus. During this monitoring hiatus, BTV-5, BTV-7, and BTV-24 were first isolated in China in 2012–2014, demonstrating that BTV continues to evolve rather than remain static in southern China. The identification of population fluctuations in BTV-16 in this study confirms this inference—without continuous monitoring, population expansions or bottleneck events occurring during the interruption period would be completely overlooked, yet these dynamics are crucial for understanding the patterns of periodic outbreaks of serotypes in Yunnan and for assessing future epidemic risks in this ecologically sensitive region.

Bayesian phylogeographic reconstruction of the BTV Seg 2 gene revealed that the four BTV serotypes circulating in Yunnan Province in this study have distinctly different geographic origins. High Bayesian factors and posterior probabilities support the origin of BTV-1 in Europe, South Asia (India), and Oceania (Australia). Moderately high Bayesian factors and posterior probabilities support the origin of BTV-4 in Oceania. Meanwhile, BTV-5 and BTV-16 are associated with transmission pathways in Oceania (Australia) and East Asia (Japan), respectively; however, the Bayesian factors and posterior probabilities supporting these origins are relatively low. In fact, this phenomenon is consistent with the phylogenetic tree, as BTV strains from Yunnan cluster together with those from Oceania (Australia). Li et al. found that BTV-16 strains isolated in Yunnan Province, China, belong to the Oriental lineage and show the highest nucleotide sequence identity (97.52%) with Japanese strains [40]. Data from four decades of continuous monitoring at Beatrice Hill Farm in northern Australia indicate that the introduction of BTV is believed to have occurred primarily through wind-borne transmission by Culicoides midges from Southeast Asia [41], rather than relying on live animal trade. Yunnan Province’s unique geographical location—bordering Myanmar, Laos, and Vietnam, and situated at the crossroads of Southeast Asia, South Asia, and East Asia—makes it a natural gateway for the introduction of viral lineages from multiple continents. At the same time, Yunnan’s hot and humid climate provides a suitable habitat for Culicoides midges, further facilitating viral establishment and reassortment. It is worth noting that the posterior probability support for the transmission pathways of BTV-5 and BTV-16 is relatively low (40–60%). This is partly due to the extremely limited number of BTV-5 sequences in global databases (only 17 were included in this study), while the East Asian transmission pathway of BTV-16 also lacks higher-confidence support due to a lack of historical data. Alkhamis et al. used a Bayesian discrete-system geographical model to reconstruct the transmission history of BTV across different geographical scales [31]. Based on an analysis of 560 Segment 10 sequences from around the world, this study indicates that intercontinental transmission of BTV dates back as far as the 1500s, and Bayesian factor analysis suggests that Europe is the most important region in the global BTV transmission network. The aim was to reveal, at a macro scale, the global origin of BTV as a viral species and the patterns of its cross-species transmission. In contrast, this study conducted phylogeographic reconstructions separately for each serotype (BTV-1, BTV-4, BTV-5, and BTV-16). This strategy enabled us to uncover the distinct transmission pathways of each serotype.

Although this study has revealed the evolutionary characteristics and transmission networks of the Bluetongue virus in Yunnan Province from the perspectives of systematic geography and population dynamics, certain limitations remain. This study relied solely on the Seg-2 gene as a molecular marker. Although the VP2 protein encoded by this segment is a core determinant of BTV serotype specificity and has been widely validated for serotype identification and phylogenetic analysis, BTV is a segmented double-stranded RNA virus, and one of the most significant genetic events in its evolution is segment reassortment under conditions of mixed infection with different strains. Therefore, the transmission pathways constructed in this study reflect only the phylogenetic history of the Seg-2 segment and may not represent the true evolutionary trajectories of other segments of the viral genome. Furthermore, because the previously published sequence data for Yunnan Province and neighboring countries obtained from the database in this study were extremely scarce, this may affect the reliability of the conclusions regarding the interregional transmission patterns of BTV. Consequently, future surveillance efforts should integrate whole-genome viral data, vector population dynamics, and information on cross-border animal movements to establish a multidimensional early warning system for bluetongue disease, thereby addressing this transboundary animal disease with significant economic implications.

## 5. Conclusions

The BTV serotypes circulating in Yunnan Province exhibit high diversity. Population dynamics vary significantly among different serotypes. These differences reflect the divergence of each serotype in terms of host adaptability, vector transmission efficiency, and local epidemiological patterns. Genomic geography based on BTV Seg 2 suggests that BTV in Yunnan may have originated from multiple sources, including Europe, South Asia, Oceania, and East Asia. Future surveillance efforts should integrate more genomic virus data, vector population dynamics, and information on cross-border animal movements to establish a multidimensional early-warning system for bluetongue, thereby effectively addressing this cross-border animal disease with significant economic implications.

## Data Availability

The nucleotide sequences of the second fragments of BTV-1, BTV-4, BTV-5, and BTV-16 described in this study have been submitted to GenBank under the accession numbers PZ244763, PZ244793, PZ244773, and PZ244783, respectively. Additional data supporting the findings of this study are available upon request from the corresponding author.

## Supplementary Materials

The following supporting information can be downloaded at: https://www.mdpi.com/article/10.3390/microorganisms14081697/s1, Table S1: BTV Seg–2 Gene Information Table.

## Abbreviations

AIC	Akaike Information Criterion
AICc	Akaike Information Criterion with correction
BIC	Bayesian Information Criterion
BT	Bluetongue
BTV	Bluetongue Virus
NCBI	National Center for Biotechnology Information
MCC	Maximum Clade Confidence
ML	Maximum Likelihood
WOAH	World Organisation for Animal Health
Seg-2	Segment 2
95%HPD	95% Highest Posterior Density
