# Supplementary material for: Molecular Epidemiological Characteristics and Genetic Evolution of Bluetongue Virus in Yunnan Province, China
Source: Microorganisms. 2026 Aug 2;14(8):1697. doi: 10.3390/microorganisms14081697 (PMC13515481; doi:10.3390/microorganisms14081697)
Supplement: Supplementary file 1 [file microorganisms-14-01697-s001.zip › microorganisms-4408135-supplementary Table S1.pdf]

Genbank	Serotype	Country	Location	Date	Host
KF664124.1	BTV-1	India	South Asia	2008	Sheep
KP339135.1	BTV-1	India	South Asia	2010	Sheep
KP339145.1	BTV-1	India	South Asia	2010	Sheep
KF563934.1	BTV-1	India	South Asia	2007	Sheep
PV939495.1	BTV-1	United Arab Emirates	West Asia	2020	Sheep
KF664114.1	BTV-1	India	South Asia	2007	Culicoides
KY934047.1	BTV-1	India	South Asia	2012	Sheep
JN558349.1	BTV-1	India	South Asia	2007	Culicoides
KF563929.1	BTV-1	India	South Asia	1985	Sheep
KF563930.1	BTV-1	India	South Asia	1994	Sheep
KF563932.1	BTV-1	India	South Asia	2003	Sheep
KP735614.1	BTV-1	India	South Asia	2011	Cattle
KF563931.1	BTV-1	India	South Asia	1999	Sheep
JN558348.1	BTV-1	India	South Asia	2007	Culicoides
KP821007.1	BTV-1	Greece	Europe	2001	Sheep
JN635334.1	BTV-1	Greece	Europe	2001	Sheep
PZ244763	BTV-1	China_Yunan(Jinghong)	Study sample	2025	Culicoides
KC879616.1	BTV-1	China_Yunan	China	1979	Sheep
KM099539.1	BTV-1	Australia	Oceania	2010	Cattle
MT212637.1	BTV-1	Australia	Oceania	2014	Cattle
MT212657.1	BTV-1	Australia	Oceania	2018	Cattle
MT212647.1	BTV-1	Australia	Oceania	2017	Cattle
MN698752.1	BTV-1	Australia	Oceania	2008	Cattle
KM099536.1	BTV-1	Australia	Oceania	2005	Cattle
KM099535.1	BTV-1	Australia	Oceania	2004	Cattle
KM099534.1	BTV-1	Australia	Oceania	2002	Cattle
KM099530.1	BTV-1	Australia	Oceania	1997	Cattle
KM099529.1	BTV-1	Australia	Oceania	1996	Cattle
KM099527.1	BTV-1	Australia	Oceania	1993	Cattle
KM099528.1	BTV-1	Australia	Oceania	1994	Cattle
KM099531.1	BTV-1	Australia	Oceania	1999	Cattle
KM099526.1	BTV-1	Australia	Oceania	1989	Cattle
MN714724.1	BTV-1	Australia	Oceania	2019	Cattle
MN698762.1	BTV-1	Australia	Oceania	2012	Cattle
KM099525.1	BTV-1	Australia	Oceania	1988	Cattle
KM099524.1	BTV-1	Australia	Oceania	1986	Cattle
KM099523.1	BTV-1	Australia	Oceania	1981	Cattle
JN881986.1	BTV-1	Australia	Oceania	1979	Cattle
MT070930.1	BTV-1	South Africa	Africa	2014	Sheep
KP821017.1	BTV-1	South Africa	Africa	1958	Sheep
MT119854.1	BTV-1	South Africa	Africa	1960	Sheep
KP821022.1	BTV-1	Sudan	Africa	1987	Cattle
OM502353.1	BTV-1	Israel	West Asia	2019	Cattle
JN848760.1	BTV-1	China_Yunan(Shizhong)	China	1997	Sheep
KP820998.1	BTV-1	Cameroon	Africa	1982	Sheep

FJ437557.1	BTV-1	France	Europe	2007	Sheep
JX861489.1	BTV-1	France	Europe	2007	Cattle
KP821021.1	BTV-1	Spain	Europe	2009	Cattle
KP821020.1	BTV-1	Spain	Europe	2008	Cattle
KP821019.1	BTV-1	Spain	Europe	2007	Cattle
KP821003.1	BTV-1	France	Europe	2008	Cattle
KJ577115.1	BTV-1	Italy	Europe	2010	Sheep
EU498674.1	BTV-1	Portugal	Europe	2007	Sheep
KP821023.1	BTV-1	Tunisia	Africa	2007	Sheep
KJ736002.1	BTV-1	Italy	Europe	2006	Sheep
KP821009.1	BTV-1	Morocco	Africa	2006	Sheep
EU625361.1	BTV-1	Algeria	Africa	2006	Sheep
EU625362.1	BTV-1	Morocco	Africa	2006	Sheep
KP820996.1	BTV-1	Algeria	Africa	2006	Sheep
KP821004.1	BTV-1	United_Kingdo	Europe	2007	Sheep
KP820997.1	BTV-1	Algeria	Africa	2008	Sheep
KJ577125.1	BTV-1	Italy	Europe	2013	Sheep
KJ577095.1	BTV-1	Italy	Europe	2012	Sheep
KJ577105.1	BTV-1	Tunisia	Africa	2011	Sheep
KP821016.1	BTV-1	Morocco	Africa	2009	Sheep
KP821008.1	BTV-1	Libya	Africa	2007	Sheep
PV295875.1	BTV-1	France	Europe	2024	Cattle
PV927958.1	BTV-1	France	Europe	2024	Sheep
KX164020.1	BTV-1	USA	North America	2010	Sheep
OP185855.1	BTV-1	France	Europe	2016	Cattle
OP185815.1	BTV-1	France	Europe	2015	Cattle
MT879212.1	BTV-4	Greece	Europe	2014	sheep
MT879200.1	BTV-4	Kosovo	Europe	2014	sheep
MT879199.1	BTV-4	Serbia	Europe	2014	cattle
MT879197.1	BTV-4	Bulgaria	Europe	2014	sheep
MT879196.1	BTV-4	Romania	Europe	2014	cattle
MT879202.1	BTV-4	Romania	Europe	2020	sheep
PV615103.1	BTV-4	Italy	Europe	2024	sheep
PV926177.1	BTV-4	Italy	Europe	2021	sheep
PV926265.1	BTV-4	Italy	Europe	2016	cattle
PP982460.1	BTV-4	Serbia	Europe	2016	cattle
PV939465.1	BTV-4	Croatia	Europe	2014	sheep
PV939475.1	BTV-4	Italy	Europe	2014	sheep
PV926207.1	BTV-4	Italy	Europe	2021	sheep
PV926197.1	BTV-4	Italy	Europe	2021	cattle
PV755179.1	BTV-4	Tunisia	Africa	2020	cattle
PV974506.1	BTV-4	Tunisia	Africa	2019	sheep
PV076075.1	BTV-4	Spain	Europe	2021	sheep
PV615113.1	BTV-4	Italy	Europe	2024	cattle
PV740649.1	BTV-4	Tunisia	Africa	2021	cattle
PV926245.1	BTV-4	Morocco	Africa	2014	sheep

MT043259.1	BTV-4	South Africa	Africa	2017	sheep
KT317666.1	BTV-4	South Africa	Africa	2011	sheep
KP821071.1	BTV-4	Sudan	Africa	1983	cattle
PV956183.1	BTV-4	Italy	Europe	2012	sheep
PX442672.1	BTV-4	Spain	Europe	2024	sheep
KP821068.1	BTV-4	Spain	Europe	2004	sheep
KP821061.1	BTV-4	Morocco	Africa	2004	sheep
KP821069.1	BTV-4	Spain	Europe	2005	cattle
EF434176.1	BTV-4	Portugal	Europe	2004	sheep
DQ191281.1	BTV-4	Italy	Europe	2003	Culicoides
KP821067.1	BTV-4	Spain	Europe	2003	sheep
JN255893.1	BTV-4	Italy	Europe	2003	sheep
KP821070.1	BTV-4	Spain	Europe	2010	cattle
KP821064.1	BTV-4	Morocco	Africa	2009	sheep
KP821059.1	BTV-4	Israel	West Asia	2006	sheep
OQ398245.1	BTV-4	Israel	West Asia	2015	sheep
DQ191277.1	BTV-4	Greece	Europe	1999	sheep
KP821051.1	BTV-4	Greece	Europe	1979	sheep
KP821040.1	BTV-4	Cyprus	West Asia	1969	cattle
KP821054.1	BTV-4	Greece	Europe	2000	sheep
KP821041.1	BTV-4	Cyprus	West Asia	2004	sheep
KP821058.1	BTV-4	Greece	Europe	1978	sheep
KP821044.1	BTV-4	Cyprus	West Asia	2011	sheep
KP821057.1	BTV-4	Greece	Europe	2012	sheep
JX024960.1	BTV-4	Argentina	South America	2010	cattle
JX024955.1	BTV-4	Argentina	South America	2009	cattle
JX024940.1	BTV-4	Argentina	South America	1999	cattle
MT070950.1	BTV-4	South Africa	Africa	1960	sheep
MT070920.1	BTV-4	South Africa	Africa	2014	sheep
KY947351.1	BTV-4	India	West Asia	2013	sheep
KY947343.1	BTV-4	India	West Asia	2007	sheep
KY947345.1	BTV-4	India	West Asia	2012	sheep
KF560418.1	BTV-4	India	West Asia	2008	sheep
PZ244793	BTV-4	China_Yunan(Shizhong)	Study sample	2025	Culicoides
MN714844.1	BTV-4	Australia	Oceania	2014	cattle
MN714804.1	BTV-4	Australia	Oceania	2006	cattle
MN714834.1	BTV-4	Australia	Oceania	1999	cattle
MN714824.1	BTV-4	Australia	Oceania	1996	cattle
JX560414.1	BTV-4	China_Yunnan	China	1997	cattle
MT070940.1	BTV-5	South Africa	Africa	1953	sheep
KP821101.1	BTV-5	South Africa	Africa	1953	sheep
JX272570.1	BTV-5	South Africa	Africa	2012	NA
MT070880.1	BTV-5	South Africa	Africa	2014	sheep
MZ215862.1	BTV-5	South Africa	Africa	1953	NA
AJ585181.1	BTV-5	Cameroon	Africa	1982	NA
MT043249.1	BTV-5	South Africa	Africa	2017	sheep

MG924987.1	BTV-5	Australia	Oceania	2015	cattle
KT945045.1	BTV-5	China_Yunnan	China	2012	cattle
KT945044.1	BTV-5	China_Yunnan	China	2012	cattle
KT945042.1	BTV-5	China_Yunnan	China	2012	cattle
KT945043.1	BTV-5	China_Yunnan	China	2014	cattle
PZ244773	BTV-5	China_Yunnan(Jinghong）	Study sample	2025	Culicoides
PX460303.1	BTV-5	Italy	Europe	2025	sheep
AJ585182.1	BTV-5	Nigeria	Africa	1982	NA
KX164060.1	BTV-5	America	North America	2003	cattle
HQ241072.1	BTV-5	France	Europe	2010	cattle
LC586239.1	BTV-16	Japan	East Asia	2014	cattle
AB686226.1	BTV-16	Japan	East Asia	2008	Culicoides
AB686225.1	BTV-16	Japan	East Asia	2002	Culicoides
AB686222.1	BTV-16	Japan	East Asia	2002	cattle
AB686220.1	BTV-16	Japan	East Asia	2001	Culicoides
AB686221.1	BTV-16	Japan	East Asia	2001	Culicoides
MK014493.1	BTV-16	Turkey	Europe	2010	sheep
KP820992.1	BTV-16	Israel	West Asia	2008	sheep
KP306782.1	BTV-16	Israel	west Asia	2008	sheep
KP820986.1	BTV-16	Cyprus	West Asia	2006	cattle
KP306779.1	BTV-16	Israel	west Asia	2011	cattle
KP820987.1	BTV-16	Cyprus	West Asia	2010	cattle
KP820985.1	BTV-16	Cyprus	West Asia	2004	sheep
KP306781.1	BTV-16	Israel	west Asia	2013	cattle
AJ585148.1	BTV-16	Turkey	Europe	2000	NA
KP306785.1	BTV-16	Israel	west Asia	2008	cattle
KP306780.1	BTV-16	Israel	west Asia	2013	sheep
KP306778.1	BTV-16	Israel	west Asia	2012	cattle
KP820990.1	BTV-16	Greece	Europe	2008	sheep
KP820989.1	BTV-16	Greece	Europe	1999	sheep
AM773709.1	BTV-16	Greece	Europe	1999	NA
AM773702.1	BTV-16	Greece	Europe	1999	NA
KP820994.1	BTV-16	Turkey	Europe	2000	NA
AJ585146.1	BTV-16	Turkey	Europe	2000	NA
AJ585147.1	BTV-16	Turkey	Europe	2000	NA
KF664134.1	BTV-16	India	South Asia	2011	sheep
KY934050.1	BTV-16	India	South Asia	2012	sheep
KY934051.1	BTV-16	India	South Asia	2013	sheep
KF664104.1	BTV-16	India	South Asia	2008	sheep
JQ924821.1	BTV-16	India	South Asia	2010	sheep
KP339215.1	BTV-16	India	South Asia	2011	sheep
KP339205.1	BTV-16	India	South Asia	2010	sheep
KP339195.1	BTV-16	India	South Asia	2010	sheep
MG710529.1	BTV-16	India	South Asia	2007	NA
MG710531.1	BTV-16	India	South Asia	2017	NA
KP339225.1	BTV-16	India	South Asia	2010	sheep

KX302635.1	BTV-16	India	South Asia	2014	sheep
ON675589.1	BTV-16	India	South Asia	2016	sheep
JX007924.1	BTV-16	India	South Asia	2010	cattle
JN572917.1	BTV-16	India	South Asia	2008	sheep
MG710530.1	BTV-16	India	South Asia	2017	NA
MH999880.1	BTV-16	Italy	Europe	2007	cattle
MH990404.1	BTV-16	Italy	Europe	2006	NA
MH990434.1	BTV-16	Italy	Europe	2004	sheep
MH990424.1	BTV-16	Italy	Europe	2004	sheep
DQ191259.1	BTV-16	Israel	west Asia	2005	NA
JX272460.1	BTV-16	South Africa	Africa	2012	NA
AM773700.1	BTV-16	Italy	Europe	2004	NA
AM773699.1	BTV-16	Italy	Europe	2004	NA
KF387522.1	BTV-16	Italy	Europe	2002	cattle
DQ191260.1	BTV-16	Italy	Europe	2001	NA
MH990414.1	BTV-16	Italy	Europe	2007	sheep
KP820988.1	BTV-16	France	Europe	2004	sheep
KP820993.1	BTV-16	Pakistan	South Asia	1960	sheep
AJ585149.1	BTV-16	South Africa	Africa	2003	NA
KP820991.1	BTV-16	Israel	west Asia	2001	sheep
AM773710.1	BTV-16	Israel	west Asia	2001	NA
AM773701.1	BTV-16	Israel	west Asia	2001	NA
AJ585137.1	BTV-16	South Africa	Africa	2003	NA
AB686217.1	BTV-16	Japan	East Asia	1990	cattle
AB686214.1	BTV-16	Japan	East Asia	1987	cattle
AB686213.1	BTV-16	Japan	East Asia	1985	cattle
PZ244783	BTV-16	China_Yunnan(Shuangjiang）	Study sample	2025	Culicoides
JQ086232.1	BTV-16	Australia	Oceania	1987	cattle
MN695909.1	BTV-16	Australia	Oceania	2016	cattle
MN227138.1	BTV-16	Australia	Oceania	1992	cattle
MN747851.1	BTV-16	Australia	Oceania	2000	sheep
AJ585151.1	BTV-16	Indonesia	Oceania	1991	NA
MN714784.1	BTV-16	Papua New Guinea	Oceania	2016	cattle
MN747871.1	BTV-16	Australia	Oceania	2017	cattle
MN714774.1	BTV-16	Australia	Oceania	2017	cattle
MT212667.1	BTV-16	Australia	Oceania	2017	cattle
MN714764.1	BTV-16	Australia	Oceania	2018	cattle
MN714754.1	BTV-16	Australia	Oceania	2018	cattle
MN714744.1	BTV-16	Australia	Oceania	2016	cattle
MT212677.1	BTV-16	Australia	Oceania	2019	cattle
MN747881.1	BTV-16	Australia	Oceania	2017	cattle
MN747861.1	BTV-16	Australia	Oceania	2018	cattle
MT078350.1	BTV-16	South Africa	Africa	1971	sheep
AJ585150.1	BTV-16	Nigeria	Africa	1982	NA
